# Resistance of oat breeding lines to grain contamination with Fusarium langsethiae and T-2/HT-2 toxins

**DOI:** 10.18699/VJ21.083

**Published:** 2021-11

**Authors:** O.P. Gavrilova, T.Yu. Gagkaeva, A.S. Orina, A.S. Markova, A.D. Kabashov, I.G. Loskutov

**Affiliations:** All-Russian Institute of Plant Protection, Pushkin, St. Petersburg, Russia; All-Russian Institute of Plant Protection, Pushkin, St. Petersburg, Russia; All-Russian Institute of Plant Protection, Pushkin, St. Petersburg, Russia; Federal Research Center “Nemchinovka”, Novoivanovskoe, Moscow region, Russia; Federal Research Center “Nemchinovka”, Novoivanovskoe, Moscow region, Russia; Federal Research Center the N.I. Vavilov All-Russian Institute of Plant Genetic Resources (VIR), St. Petersburg, Russia

**Keywords:** Avena sativa, naked, breeding, resistance, Fusarium, DNA, mycotoxins, Avena sativa, голозерный, селекция, устойчивость, фузариоз, ДНК, микотоксины

## Abstract

Fusarium disease of oats reduces yield quality due to decreasing germination that is caused by the contamination of grain with mycotoxins produced by Fusarium fungi. The aim of this study was to characterize the resistance of naked breeding lines of oats to fungal grain infection and to contamination with T-2 and HT-2 toxins. Thirteen naked oat breeding lines and two naked varieties, Nemchinovsky 61 and Vyatskiy, as well as a husked variety Yakov, were grown under natural conditions in the Nemchinovka Federal Research Center in 2019–2020. The contamination of grain with fungi was determined by the mycological method and real-time PCR. The analysis of mycotoxins was carried out by ELISA. In oats, Alternaria (the grain infection was 15–90 %), Cochliobolus (1–33 %), Cladosporium (1–19 %), Epicoccum (0–11 %), and Fusarium (3–17 %) fungi prevailed in the grain mycobiota. The predominant Fusarium species were F. poae (its proportion among Fusarium fungi was 49–68 %) and F. langsethiae (29–28 %). The highest amounts of F. langsethiae DNA ((27.9–71.9) × 10–4 pg/ng) and T-2/HT-2 toxins (790–1230 μg/kg) were found in the grain of husked oat Yakov. Among the analysed naked oat lines, the amount of F. langsethiae DNA varied in the range of (1.2–42.7) × 10–4 pg/ng,and the content of T-2/HT-2 toxins was in the range of 5–229 μg/kg. Two oat breeding lines, 54h2476 and 66h2618, as well as a new variety, Azil (57h2396), can be characterized as highly resistant to infection with Fusarium fungi and contamination with mycotoxins compared to the control variety Vyatskiy.

## Abstract (ru)

Фузариоз относится к важным заболеваниям овса, поскольку многие виды грибов Fusarium способны продуцировать микотоксины, негативно влияющие на качество зерна. Иммунных к заражению грибами Fusarium зерновых культур нет, однако наблюдаются различия генотипов по степени устойчивости. Целью исследования стала характеристика перспективных линий голозерного овса по устойчивости к зараженности зерна грибами и содержанию фузариотоксинов. Анализировали 13 селекционных линий и два сорта голозерного овса, Немчиновский 61 и Вятский, а также сорт пленчатого овса Яков, которые выращивали на естественном фоне в Федеральном исследовательском центре «Немчиновка» в 2019–2020 гг. Зараженность зерна грибами определяли микологическим методом, а также с помощью полимеразной цепной реакции (ПЦР) в реальном времени, анализ микотоксинов выполняли иммуноферментным методом. Проведенные анализы показали, что зерно всех образцов овса было заражено грибами, однако их численность и видовое разнообразие варьировали в зависимости от анализируемого генотипа и года исследований. Микобиоту генотипов овса преимущественно составляли виды родов Alternaria (15–90 % от всех выделенных грибов), Cochliobolus (1–33 %), Cladosporium (1–19 %), Epicoccum (0–11 %) и Fusarium (3–17 %). Основными представителями фузариевых грибов были F. poae, продуцирующий ниваленол, и F. langsethiae, производящий Т-2/НТ-2 токсины. Наибольшее количество ДНК F. langsethiae, а также Т-2/ НТ-2 токсинов содержалось в зерне пленчатого сорта Яков и составило (27.9–71.9) × 10–4 пг/нг и 790– 1230 мкг/ кг соответственно. В зерне анализируемых линий овса содержание ДНК F. langsethiae варьировало в диапазоне (1.2– 42.7) × 10–4 пг/нг, Т-2/НТ-2 токсинов – от 5 до 229 мкг/кг. Две линии голозерного овса, 54h2476 и 66h2618, а также новый сорт Азиль (линия 57h2396) можно охарактеризовать в условиях эксперимента как высокоустойчивые к заражению грибами Fusarium и контаминации микотоксинами в сравнении с контролем – сортом Вятский.

## Introduction

Over the past decade, the amount of information on Fusarium disease of oats (Avena sativa L.) has increased dramatically. The infection of oats caused by different Fusarium Link species is recognized as one of the most devastating diseases of this cereal crop. In addition to direct negative impacts on economically valuable traits, such as the loss of grain yield (Martinelli et al., 2014), the harmfulness of Fusarium fungi is determined by their ability to produce different mycotoxins that accumulate in infected grains. Mycotoxins produced by many Fusarium species remain in processed products and, when consumed by people or animals, can cause immunosuppression and various health issues (Foroud et al., 2019). Current studies of the Fusarium problem in oats concern the analysis of grain infection by different fungal species and the determination of mycotoxin contents in grain (Fredlund et al., 2013; Gavrilova et al., 2016; Hofgaard et al., 2016; Schöneberg et al., 2018), the study of host-pathogen interactions (Divon et al., 2012; Tekle et al., 2012; Martin et al., 2018; Wilforss et al., 2020) and the search for potential sources of resistance to the disease, including the use of molecular analysis methods (He et al., 2013; Bjørnstad et al., 2017; Isidro-Sánchez et al., 2020).

The composition and representation of Fusarium species causing the disease in oats vary significantly and depend on the place of cultivation and the prevailing weather conditions during the growing season (Schöneberg et al., 2018). As a rule, the main species of Fusarium fungi responsible for disease in oats are F. poae (Peck) Wollenw., F. sporotrichioides Sherb. and F. langsethiae Torp & Nirenberg (Kurowski, Wysocka, 2009; Fredlund et al., 2013; Gavrilova et al., 2016; Hofgaard et al., 2016), while F. graminearum Schwabe (Schöneberg et al., 2018) and F. avenaceum (Fr.) Sacc. (Vargach et al., 2019) occur less often. All of the mentioned Fusarium fungi are capable of producing various mycotoxins. The results of numerous studies demonstrate a high contamination of grain with T-2 and HT-2 toxins produced by F. sporotrichioides and F. langsethiae (Opoku et al., 2013; Burkin et al., 2015; Hofgaard et al., 2016; Kononenko et al., 2020; De Colli et al., 2021).

In the breeding of oat varieties, the trait of resistance to Fusarium disease was not taken into account for a long time despite the problem with grain infection of this cereal crop. The main challenge of the evaluation of resistance of oat genotypes to the disease in the field is the absence or weak symptoms of Fusarium infection on oat panicles, in contrast to the noticeable specific symptoms on heads of other smallgrain cereals (Tekauz et al., 2008; Imathiu et al., 2013; Martin et al., 2018; Zhuikova, Batalova, 2019). However, Fusarium fungi and mycotoxins in the grain of asymptomatic spikelets in panicles are often detected, and oat genotypes can be significantly different according to their amounts. In addition, it is already well known that the disease severity is determined by factors such as the weather and infection pressure.

There are no cereals that are immune to infection with Fusarium fungi; however, different degrees of resistance are observed among genotypes. Previously, it was mentioned that a wheat genotype resistant to infection with one Fusarium species also tends to be resistant to other species of this genus (Mesterhazy et al., 2005). Additionally, several types of resistance to Fusarium disease in cereals have been described and commonly divided into at least five separate types (Boutigny et al., 2008; Tekle et al., 2018): resistance against initial infection (type I), resistance against the spread of infection (II), resistance against grain infections (III), tolerance (IV), and resistance to mycotoxin accumulation or degradation (V). In the sowing oats (A. sativa L.), two subspecies, husked oats (A. sativa subsp. sativa L.) and naked oats (A. sativa subsp. nudisativa (Husn.) Rod. et Sold.), which differ from each other in their morphological characteristics, biochemical properties and resistance to abiotic and biotic factors, were described (Kobylyansky, Soldatov, 1994; Loskutov et al., 2020). The relatively high resistance of naked oats to Fusarium infection of grain, in comparison with husked oats, has been repeatedly noted (Tekauz et al., 2008; Yan et al., 2010; Gagkaeva et al., 2013; Martin et al., 2018; Chropová et al., 2020).

Earlier, information on the resistance of oat genotypes from the VIR collection to Fusarium disease, which was analysed under conditions of artificial inoculation with F. sporotrichioides, was systematized in the Catalogue (Gagkaeva et al., 2012). A successful example of combining the efforts of different research groups was the breeding of a new variety of naked oats, Vsadnik, which is the first officially registered variety in Russia characterized as relatively resistant to Fusarium disease. This variety accumulated significantly lower amounts of mycotoxins in the grain than the standard husked variety Konkur, which is cultivated over a wide area in Russia (Mishenkina, Zakharov, 2017).

At present, the attention of many Russian oat breeders is focused on the creation of naked oat varieties characterized by improved grain quality and resistance to fungal diseases (Kabashov et al., 2018; Batalova et al., 2019; Isachkova et al., 2019; Zhuikova et al., 2020). The progress achieved in the breeding process is evidenced by the increase in the number of naked oat varieties included in the “State Register of Selection Achievements…”, which in 2020 consisted of 121 varieties of husked oats and 15 varieties of naked oats1. ( 1 The State Register of Selection Achievements Approved for Use. Vol. 1. Plant Varieties (at February 26, 2020) https://gossortrf.ru/gosreestr// ) Since 2010, 11 new varieties of naked oats have been included in the State Register.

The aim of this study was to characterize the resistance of naked oat lines to contamination of grain with Fusarium fungi and T-2/HT-2 toxins. These oat genotypes are the breeding material of the Federal Research Center “Nemchinovka” and were cultivated in field experiments under natural conditions.

## Materials and methods

Oats breeding material. 10 and 13 naked oat breeding lines (A. sativa subsp. nudisativa (Husn.) Rod. et Sold.) were analysed in 2019 and 2020, respectively. In addition, the naked oat varieties Nemchinovsky 61 (NFRC) and Vyatsky as a control (Zonal North-East Agricultural Research Institute, Kirov region) and the standard husked variety Yakov (NFRC) were included in the study (Table 1).

**Table 1.**
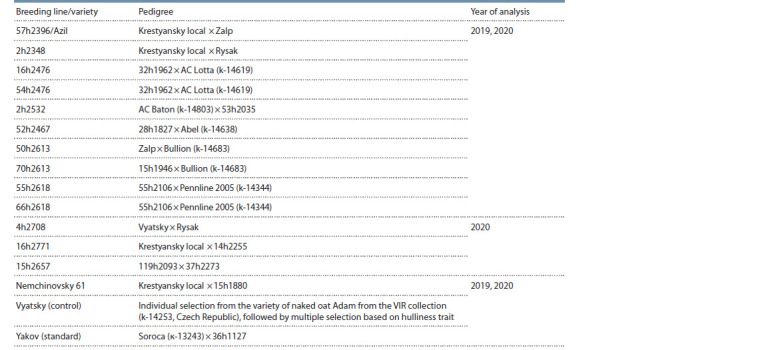
The breeding lines and varieties of oats included in the study

Cultivation of breeding material. In 2019‒2020, the analysed varieties and breeding lines of oats were grown after spring barley as the previous crop in the experimental 10 m2 plots in the nursery of the NFRC according to the state variety testing methodology2.( Methodology for State Variety Testing of Agricultural Crops. Second edition. Grains, Cereals, Legumes, Corn and Fodder Crops. Moscow, 1989.) The harvesting of oats was carried out at the full-mature stage: August 8, 2019, and August 16,2020. The weather conditions in the growing seasons of 2019 and 2020 were different (Table 2). The summer period of 2020 was characterized by an increased temperature in June- August compared to the long-term average values, as well as a 1.7‒2.6 times excess of the total precipitation in May-July compared to this period in 2019.

**Table 2.**
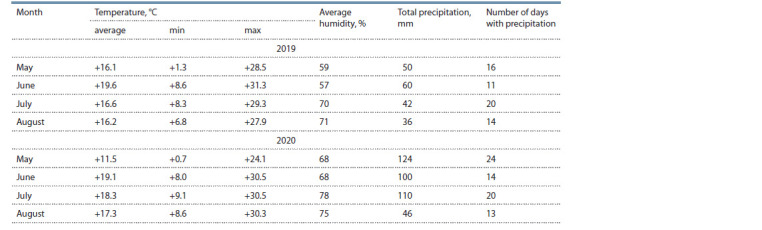
The weather conditions of summer 2019 and 2020 in the Moscow region (meteorological station No. 27515)

Mycological analysis of grain infection. To evaluate the fungal infection and species composition of oat grain mycobiota, 100 seeds of each genotype were surface sterilized in 5 % sodium hypochlorite and washed with sterilized water. Then, grains were placed on potato sucrose agar medium (PSA) in Petri dishes (Orina et al., 2018), and incubated in the dark at 24 °С in an MIR-254 thermostat (Sanyo, UK). After seven days, the number and species diversity of fungi isolated from the grain were registered.

The taxonomic status of the isolated fungi was determined by the sum of macro- and micromorphological characters according to the manuals (Ellis, 1971; Gerlach, Nirenberg, 1982; Samson et al., 2002; Torp, Nirenberg, 2004).

The grain infection by definite fungi was quantified as the percentage ratio of the number of grains from which these fungi were isolated to the total number of analysed grains.

Analysis of F. langsethiae DNA content. Ten grams of grain of every oat genotype was homogenized separately using sterilized grinding chambers of a Tube Mill Control batch mill (IKA, Germany) at 25,000 rpm for 30–45 s. Total DNA from 200 mg of grain flour was isolated using the CTAB method (Gagkaeva et al., 2013). Genomic DNA was isolated from the mycelium of a typical F. langsethiae strain from the collection of the Laboratory of Mycology and Phytopathology of All-Russian Institute of Plant Protection using a Genomic DNA Purification Kit (Thermo Fisher Scientific, Lithuania) according to the manufacturer’s protocol.

The DNA concentrations from the grain samples and from fungal strains were determined using a Qubit 2.0 fluorometer with a Quant-iT dsDNA HS Assay Kit (Thermo Fisher Scientific, USA). Before the start of quantitative PCR (qPCR), the concentrations of all DNA samples were aligned to 20‒60 ng/ μL.

The F. langsethiae DNA content in every DNA sample extracted from oat flour was estimated by qPCR with a TaqMan probe fluorescently labelled with Cy5 dye and a BHQ-2 quencher (Yli-Mattila et al., 2008).

Amplification reactions were run using the CFX 96 Real- Time System (BioRad, USA) according to the following protocol: 1 × [95 °C, 3 min]; 40 × [95 °C, 10 s; 60 °C, 10 s; 72 °C, 20 s]. The DNA content was calculated as the ratio of fungal DNA to total DNA in each sample (pg/ng).

Analysis of mycotoxin content. The mycotoxins were extracted from 1 g of oat flour with 5 mL of an acetonitrile:water mixture (84:16, v/v) for 14–16 h. The total amounts of T-2 and HT-2 toxins in the extracts were determined using an indirect competitive enzyme-linked immunosorbent assay. The diagnostic certified test system “T-2 toxin–ELISA” (All-Russian Research Institute for Veterinary Sanitation, Hygiene and Ecology, Russia) was used. The limit of mycotoxin detection was 4 μg/kg.

Statistical analysis. The contents of fungal DNA and mycotoxins in the grain of each genotype were analysed at least twice. The mean values, confidence intervals, Pearson coefficients of correlation (r) between quantitative parameters and variance analysis (ANOVA) were performed using Microsoft Excel 2010, Minitab 17 and Statistica 10.0 programs. Differences were considered significant at p < 0.05.

## Results

Fungal infection of oat grain

The predominance of fungi belonging to Alternaria Nees, Cochliobolus Drechsler, Cladosporium Link, Epicoccum Link, and Fusarium genera in the grain of the analysed oat genotypes was revealed by the mycological method. In addition, the fungi Acremonium Link, Arthrinium Kunze, Gliocladium Corda, Microdochium Syd. & P. Syd., Mucor Fresen., Nigrospora Zimm., Penicillium Link, Phoma Sacc., and Trichothecium Link genera were sporadically isolated from the grain.

**Fig. 1.**
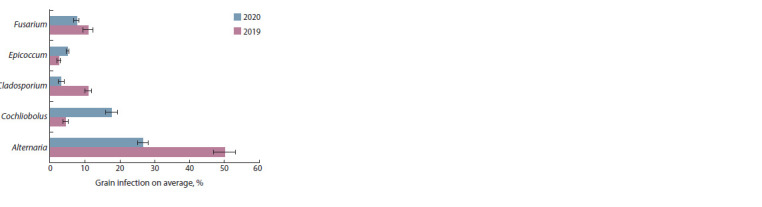
Fungal infection of oat grain (Federal Research Center “Nemchinovka”, Moscow region, 2019–2020).

Alternaria fungi were the most abundant in the oat grain mycobiota in both years of the study (Fig. 1). The majority of isolated Alternaria spp. was represented by fungi belonging to section Alternaria (86 % in 2019 and 84 % in 2020), and the remaining isolates were identified as Alternaria fungi belonging to section Infectoriae

Grain infection with Cochliobolus fungi, including Bipolaris sorokiniana Shoemaker, Drechslera avenae (Eidam) Sharif (Pyrenophora avenae Ito & Kurib) and others, differed significantly in the years of study ( p = 0.000012). In 2019, oat grain infection with Cochliobolus spp. varied in the range of 1–13 %, whereas in 2020, its incidence was 6–33 %.

The proportion of grains colonized by Fusarium spp. did not differ significantly between the years of study. The incidences of Fusarium infection of grain of naked oat lines and the variety Nemchinovsky 61 varied from 5 to 17 % in 2019 and from 3 to 13 % in 2020. For the husked variety Yakov, the incidences of Fusarium grain infection were 26 and 17 % in 2019 and 2020, respectively. For the control variety Vyatsky, the incidences were 5 and 3 %, respectively. In both years of the study, only two lines, 54h2476 and 66h2618, were characterized by lower grain infection or coincidence with the control variety Vyatsky grain infection with Fusarium spp. At least nine Fusarium species were identified in the mycobiota of oat grains, but toxin-producing F. poae and F. langsethiae species prevailed in both years. The proportions of these fungi among Fusarium spp. isolates were 49‒68 % for F. poae and 29‒28 % for F. langsethiae.

Contents of F. langsethiae DNA and Т-2/НТ-2 toxins

**Fig. 2.**
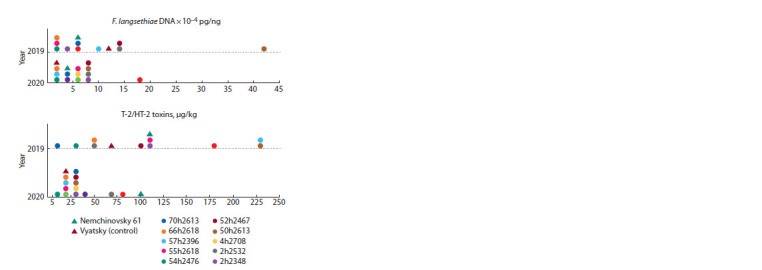
Contents of F. langsethiae DNA and T-2/HT-2 toxins in the grain of the varieties and breeding lines of oats (Federal Research Center “Nemchinovka”).

The highest content of F. langsethiae DNA was revealed in the grain of the husked variety Yakov and amounted to 71.9 × 10–4 pg/ng in 2019 and 27.9 × 10–4 pg/ng in 2020. In the grain of the control naked variety Vyatsky, the F. langsethiae DNA content was significantly lower, reaching 11.0 × 10–4 pg/ ng in 2019 and 1.2 × 10–4 pg/ng in 2020. In 2019, only three oat breeding lines, 2h2532, 52h2467, and 50h2613, contained more F. langsethiae DNA than the control variety Vyatsky. In 2020, the contents of fungal DNA in the grain of all analysed oat breeding lines were higher than that in the control variety (Fig. 2).

## Discussion

The mycological analysis revealed the presence of fungal infection in the grain of all oat genotypes; however, the number and species composition of identified micromycetes varied depending on oat genotype and crop year.

The average temperatures during the vegetation seasons in both years were similar; however, the precipitation in May- August in 2020 was two times higher than that observed in the previous year. As a result, the average grain infection with Cochliobolus increased fourfold in 2020; at the same time, grain infections with Alternaria, Cladosporium and Fusarium fungi significantly decreased 1.4‒3.5 fold.

With the high incidence of Cochliobolus infection of oat grain in 2020, a significant negative correlation between infection with Cochliobolus and Alternaria fungi was revealed (r = ‒0.56, р = 0.024). Previously, antagonistic relationships between these two groups of fungi associated with small-grain cereals were also established (Kazakova et al., 2016; Gannibal, 2018; Orina et al., 2020). Perhaps the Cochliobolus were more competitive in the wetter conditions, and these fungi had an advantage over Alternaria and Fusarium fungi.

Significant positive correlations between grain infection and Alternaria and Fusarium fungi (r = 0.64, р = 0.019) and Epicoccum and Fusarium fungi (r = 0.57, р = 0.043) were revealed in 2019. A symbiotic relationship between Alternaria and Fusarium fungi in cereal grain has been established repeatedly (Kosiak et al., 2004; Orina et al., 2017; Karakotov et al., 2019).

Among all Fusarium fungi isolated from oat grains, the F. poae and F. langsethiae strains were dominant. F. poae produce nivalenol and diacetoxyscirpenol and F. langsethiae is a strong producer of T-2/HT-2 toxins and DAS. In Russia, the amounts of T-2/HT-2 toxins are regulated in oat grains for food and feed, and the maximal permissible limit is 100 μg/kg3, 4.( 3 Technical Regulation of Custom Union 015/2011 “About grain safety” with changes from 15 September 2017. Supplementary 2. 4 Technical Regulation of Custom Union 021/2011 “About food safety” with changes from 8 August 2019. Supplementary 3.)

The relatively low infection of grain with F. langsethiae (with maxima of 14 and 5 % in 2019 and 2020, respectively) led to high amounts of detected mycotoxins. Therefore, we evaluated the breeding material by both the presence of F. langsethiae DNA and the accumulation of the sum of T-2 and HT-2 toxins in grain.

The highest incidence of infection with F. langsethiae and the maximal amounts of fungal DNA and T-2/HT-2 toxins were found in the grain of the husked variety Yakov. In comparison with this genotype, all naked breeding lines and varieties were less infected and contained significantly less fungal DNA and mycotoxins. Significant positive correlations between the amounts of F. langsethiae DNA and T-2/HT-2 toxins in the grain of naked oat genotypes were found (r = 0.54, p = 0.069 in 2019, and r = 0.51, p = 0.054 in 2020).

The results of our study demonstrated significant differences in oat breeding lines and varieties according to the content of F. langsethiae DNA in grain, although all genotypes were contaminated with T-2/HT-2 toxins. Thus, it is worth emphasizing again that the evaluation of oat resistance to Fusarium disease should be carried out according to several parameters.

It has been suggested that oat resistance type V to Fusarium disease depends on the mycotoxin type and that the QTLs associated with a low level of accumulation of deoxynivalenol in grain might not provide the resistance of the same genotype to other mycotoxins (He et al., 2013; Martin et al., 2018). However, comparison of the results obtained in our study (Gagkaeva et al., 2013) and later studies of the same oat genotypes under different conditions, genotype VIR-7766 (Hautsalo et al., 2021), varieties Argamak (Willforss et al., 2020) and Vyatsky (Chrpová et al., 2020), demonstrated a relatively high resistance of these oats to the accumulation of different mycotoxins, such as T-2/HT-2 toxins and deoxynivalenol.

The genetic basis of oat breeding lines plays a key role in their resistance to Fusarium disease. In the pedigree of two naked oat lines, characterized by high contents of fungal DNA and mycotoxins in 2019, the Zalp variety was recorded. Apparently, the crossing of breeding material with this variety can promote an increase in genotype susceptibility to Fusarium disease.

## Conclusion

The breeding lines of naked oats created in the Federal Research Center “Nemchinovka” were evaluated by the sum of parameters characterized by different types of oat resistance to Fusarium disease. The amounts of F. langsethiae DNA and T-2/HT-2 toxins produced by this fungus were analysed, and based on the results obtained during a two-year study, under growth conditions, two lines of naked oats, 54h2476 and 66h2618, and the new variety, Azil (Line 57h2396 in 2019), demonstrated relatively high resistance to F. langsethiae infection and mycotoxin contamination compared with the control naked variety Vyatsky. These lines should be actively used to create new varieties that do not accumulate mycotoxins and are characterized by high-quality grain.

## Conflict of interest

The authors declare no conflict of interest.
